# Assessment of quality and readability of internet-based health information related to commonly prescribed angiotensin receptor blockers

**DOI:** 10.11604/pamj.2020.35.70.18237

**Published:** 2020-03-11

**Authors:** Anuoluwapo Oloidi, Sabina Onyinye Nduaguba, Kehinde Obamiro

**Affiliations:** 1 Medplus Health and Beauty, Saka Tinubu, Lagos State, Nigeria; 2 Health Outcomes and Pharmacy Practice 'Division' College of Pharmacy, University of Texas at Austin, USA; 3 Centre for Rural Health, College of Health and Medicine, University of Tasmania, Australia

**Keywords:** Health information, readability, quality, losartan, valsartan, irbesartan

## Abstract

**Introduction:**

Hypertension is a global public health burden. Angiotensin receptor blockers (ARBs) have proven efficacy in the management of hypertension and related complications. The Internet has become a major source of health information for patients and healthcare professionals. The study aimed to assess the quality and readability of internet-based information related to selected Angiotensin Receptor Blockers (ARBs).

**Methods:**

The three most widely used ARBs were identified from published literature, after which internet-based patient information was identified from the first five pages of three search engines (Google, Yahoo and Bing). Quality of identified websites were assessed using the DISCERN instrument, while readability was evaluated using the SMOG instrument and the Flesch-Kincaid readability algorithm. Final ratings were then calculated as described by the instruments developers. Further, inter-class correlation coefficients (ICC) were calculated using the Statistical Package for Social Sciences.

**Results:**

The average overall DISCERN score in this study was 2.99 (SD±1.05). No website received an excellent rating, 15% were rated good, 66% as moderate and 19% as poor. The inter-class reliability was 0.804 for losartan and 0.695 for valsartan. The mean Flesch Reading Ease score for the websites was 48.87 (SD±16.12), mean Flesch-Kincaid Reading Grade Level was 9.29 (SD±1.98) while mean SMOG value was 11.29 (SD±1.70).

**Conclusion:**

Overall, patient information on the reviewed ARBs websites was found to be of moderate quality and suboptimal readability. Content providers on websites should ensure that health information is of favorable quality and easy to read by patients with varying degree of health literacy.

## Introduction

The internet has rapidly become the largest source of information worldwide [1] and an important source of health information both for patients and health professionals [2]. With the advent of the internet, medical information is easily accessed with the click of a mouse for several disease conditions requiring short-term or long-term therapy [3]. Studies have showed that in Canada and the United States, more than 80% of the population have access to the internet [4], and about 72% of United State users seek information over the internet [5]. Studies have also showed that health information are among the most sought after topics on the internet [6], not necessarily as a means of replacing advice given by health professionals, but for validation of information given and to gather additional information [7]. Increasingly, patients and health professionals are turning to the internet for information pertaining to health challenges and complicated therapies [8]. One of such health challenges is hypertension, which is a major risk factor for cardiovascular diseases (CVD) [9]. In a study conducted in 2013 at the Pew Research Center, 45% of adults in the United States reported that they live with one or more chronic conditions including hypertension, lung conditions, diabetes and heart disease [10]. Sixty-seven percent of those living with hypertension were internet users, and 58% of them accessed websites that provided information about a specific medical condition or problem [10]. Hypertension is a global public health burden that contributes to morbidity, mortality and healthcare cost in both developing and developed countries [11]. Approximately 25% of hypertensive patients worldwide are taking ARBs, and about 20 million people worldwide take ARBs for both hypertension and other cardiovascular conditions [12]. The effectiveness and safety of ARBs are well established [13]. However, its use has been associated with a number of adverse drug events, especially in elderly patients. Providing quality patient information and education is therefore important for safe and effective hypertension management. Despite its potential as a significant patient information resource, the internet's usefulness is often limited by the challenges associated with finding good quality information that comes from reliable and authentic sources [14]. Previous studies have reported that more than half of websites provide poor quality health information [15]. Health professionals are therefore constantly faced with patients who have been informed or misinformed by the internet [16]. As a result, clinicians, researchers, and consumers are concerned about the quality and accuracy of online health information and it is essential to assess the validity [17]. As much as disseminating health information on the internet can help improve knowledge transfer from health professionals to the population and help individuals maintain and improve their health, the rapid development of medical information on the Internet has its shortcomings which include: 1) uneven quality of medical information available on the Internet [18]; 2) difficulty in reading and understanding this information due to use of technical language [19]; and 3) potential dangers related to its erroneous and unsuitable use [20]. In addition to addressing the issue of quality, accuracy and reliability, it is also imperative to systematically assess the presentation of online health information using readability algorithms to ensure that such information is easily read and understood. We therefore aimed to assess the quality and readability of Internet-based health information related to select Angiotensin Receptor Blockers (ARBs).

## Methods

### Search strategy

#### Identification and selection of websites

We identified the three most widely used Angiotensin Receptor Blockers from published literature: losartan, irbesartan and valsartan [21]. Each of these key terms ‘losartan', ‘irbesartan', ‘valsartan' and ‘patient education' were entered into three different search engines (Google, Yahoo, Bing) chosen based on their widespread use [22], in March 2017. Only the first 50 links reported by each search engine were identified, as studies have showed that web users only visit the top 10 websites listed in search results [23].

Websites were included in the research if they were in English Language and free to access, and if they provided patient information relating to any of the search terms. Websites designed for marketing purposes evident by the presence of diverse advertisements were excluded [24]. News feeds, video feeds, abstract listings and duplicate websites were also excluded. Additionally, websites for co-formulated ARB products were excluded. The quality of identified sites was evaluated using the DISCERN questionnaire, while readability was assessed using the SMOG calculator and Flesch-Kincaid algorithm.

### Assessment of quality of information using DISCERN instrument

The DISCERN questionnaire is a valid instrument that was designed to enable patients (or health consumers) and health care providers to assess the quality of health information. This questionnaire was developed based on the input of an expert panel, health information providers and representative of patient population [25]. The DISCERN tool is freely accessible online and a downloadable version of the DISCERN instruction handbook is available from the DISCERN handbook [26]. It is suitable for anyone who utilizes or produces information about treatment choices. Its uses include an aid for individual consumers who are making decisions about treatment, a screening tool for health information providers, a checklist for authors and producers of written consumer health information, a training tool for health professionals to improve communication and shared decision-making skills. The DISCERN questionnaire, consisting of 16 questions, was used to evaluate the quality of information on the selected websites. These questions are organized into three sections [26]. Section 1 (questions 1 to 8) addressed the reliability of the publication and helps in considering whether it can be trusted as a source of information. Section 2 (questions 9 to 15) focused on the specific details of the information about treatment choices while Section 3 (question 16) assessed the overall quality rating. Each question was rated on a 5-point scale ranging from “no” to “yes". A score of “5” indicates a definite yes; a score of “2-4” indicates that the publication meets the criterion in the question to an extent; while a score of “1” indicates a definite no.

### Readability assessment

It is generally accepted that in evaluating the readability scores of written information, the use of more than one readability formula improves the reliability of the readability scores [27], hence two readability formulae; SMOG formula [28], and Flesch-Kincaid algorithm [29] were used in this study. The SMOG readability formula was created by G Harry McLaughlin in 1969 and it estimates the years of education a person needs to understand a piece of writing [28]. SMOG readability grades were measured using the manual SMOG formula [28]. The SMOG was then calculated as described by McLaughlin *et al.* [28]. The readability scores were calculated using Microsoft Office Word. The Flesch Reading Ease test rates the text on the given website on a 100-point scale. The higher the score, the easier it is to understand the document. The Flesch-Kincaid Grade Level test rates a text on a United States school grade level. A score of 8.0, for example, means that an eighth grader can understand the document.

**Statistical analysis:** standard data entry and analysis were done using a Microsoft Excel spreadsheet (edition 2013). Inter-class Correlation Coefficient (ICC) value was calculated using the Statistical Package for Social Sciences, version 23 (IBM, Armonk, New York, US).

## Results

A total of 450 websites were reviewed between April 2017 and September 2017- a total of 150 websites for each of the three search engines (Google, Yahoo and Bing). Removal of 363 duplicate websites left a total of 87 unique sites. Thirty-seven out of the 87 unique websites met the inclusion criteria and were eligible to be evaluated. The remaining 50 websites were excluded as 11 were not freely accessible, 2 had broken link and 37 did not provide information useful for patient education. The authors further streamlined the search results leaving only websites with patient information on losartan and valsartan considering they are the most frequently prescribed ARBs as reported in the literature [30]. This left a total of 24 websites for evaluation. The Inter-class reliability for losartan and valsartan was calculated to be 0.804 and 0.695, respectively (Table 1).

**Table 1:** Readability scores of ARB websites (N=24)

Name of website	Flesch ease reading	Flesch Kincaid grade level	Smog value
http://www.upmc.com/patients-visitors/education/cardiologydrugs/Pages/losartan.aspx	57.6	7.7	10.62
http://www.mayoclinic.org/drugs-supplements/losartan-oralroute/description/drg-20067341	35.6	12.57	12.16
https://patient.info/medicine/losartan-an-angiotensin-receptor-blockercozaar	59.4	8.7	10.43
http://www.rxlist.com/cozaar-drug/patient-how-to-take.html	19.4	12	11.34
https://dailymed.nlm.nih.gov/dailymed/drugInfo.cfm?setid=1bf520a2a50e-a6ae-0fdf-9be4e69729c0	22.5	12	14.98
http://www.auburnhospital.org/patient-education/hw-view.php?DOCHWID=d03821a1	53.7	9.1	11.6
http://www.wikidoc.org/index.php/Losartan_%28patient_information%29	52.4	9.1	10.43
http://rxoutreach.org/medicationmonograph/LosartanPotassium/Cozaar%C2%AE/Blood_pressure-Heart/	53.2	9.2	11.14
https://www.mskcc.org/cancer-care/patient-education/valsartan	78.6	4.8	7.43
http://www.rxlist.com/script/main/mobileartrx.asp?drug=diovan&monotype=multum&monopage=12	56.9	8.2	11.6
http://www.upmc.com/patients-visitors/education/cardiologydrugs/Pages/valsartan.aspx	66.8	6.3	8.24
http://www.mayoclinic.org/drugs-supplements/valsartan-oralroute/description/DRG-20067355	59.6	8.6	10.79
https://www.drugs.com/cdi/diovan.html	54.3	8.7	11.54
http://www.grandtraversesurgery.com/health-library/hw-view.php?DOCHWID=d04113a1	52.9	9.1	11.6
https://advancedurologicassociates.com/patient-education/hw-view.php?DOCHWID=d04113a1	53	9	11.47
http://www.rxlist.com/diovan-drug.htm	28	12	11.4
http://reference.medscape.com/drug/diovan-valsartan-342325	42.3	8.7	13.02
https://patient.info/medicine/valsartan-an-angiotensin-receptor-blockerdiovan	59.6	8.8	10.43
https://dailymed.nlm.nih.gov/dailymed/drugInfo.cfm?setid=62ee362f-3032-4f56-a900-4d2485b8759c	26.3	12	14.79
http://umm.edu/health/medical/drug-notes/notes/valsartan-by-mouth	51.5	9.1	10.28
http://www.empr.com/diovan/drug/110/	16.7	12	13.74
http://oregon-ent.com/patient-education/hw-view.php?DOCHWID=d04113a1	53.5	9	11.28
https://medlineplus.gov/druginfo/meds/a697015.html	50.8	9.8	10.93
https://www.blinkhealth.com/valsartan/info			
http://www.veteranshealthlibrary.org/MedicationsVA/121,918	68.2	6.6	9.73

### Assessment of quality of websites

**Figure 1:**
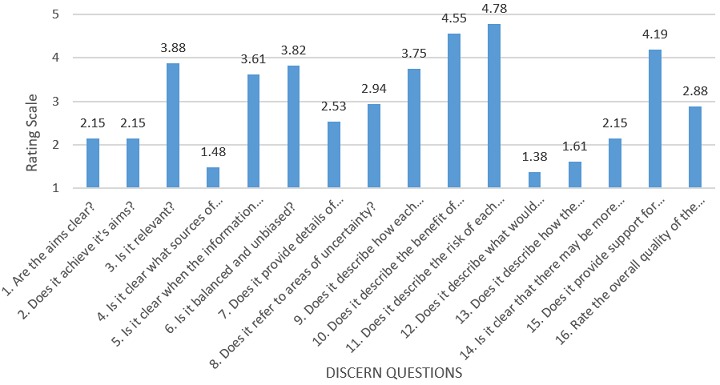
Mean quality rating of websites using the DISCERN instrument

From the evaluation conducted using the DISCERN tool, none of the websites scored 5 for question 16 (the overall quality rating at the end of the questionnaire), 15% were rated as good, 66% as moderate, while 19% were rated as poor. The overall mean DISCERN score was 2.99 (SD±1.05). The mean quality rating across the websites is shown in Figure 1. From a maximum score of 5, the mean score for the questions 1 to 8 that addressed reliability was 2.82 while for questions 9 to 15 that focused on information regarding treatment choice was 3.20. The questions with the highest response score were as follows: “Does it describe risk of each treatment?”, “Does it describe the benefit of each treatment?”, “Does it provide support for shared decision-making?” and “is it relevant?” On the other hand, the lower scoring questions are: “Is it clear what sources of information are used to compile the publication?”, “Does it describe what would happen if no treatment is used?” and “Does it describe how the treatment choice affects the overall quality of life?” The highest-rated websites according to our analysis are Mayo Clinic (Valsartan) 3.84 and Patient Info (Losartan) 3.84 [31, 32].

### Readability assessment

Twenty-four websites were assessed for readability Table 1. The mean Flesch Reading Ease Score (FRES) was 48.87 (SD±16.12), while the mean Flesch-Kincaid Reading Grade Level (FKRGL) was 9.29 (SD±1.98). The mean SMOG value was calculated to be 11.29 (SD±1.70).

## Discussion

The Internet has the potential to provide patients and health professionals with health information, but with the increase in use, concerns arise as to the quality, reliability and readability of the information obtained from the internet [33]. In addressing these concerns, several solutions have been proposed. These solutions have included electronic filtering of web-based information, creation of ethical codes of conduct for providers of web-based information (currently done on voluntary basis) and assessment of websites by health professionals [33]. Introduction of clear critical appraisal tools with standardized website evaluation systems also appears to be useful in improving the ability of users to differentiate between trustworthy sites and inadequate ones [33].

This study is the first to have systematically evaluated the quality and readability of internet-based information related the two mostly prescribed ARBs. The results from this investigation provide some insights regarding online health information related to the ARBs. The overall mean DISCERN score for the 26 websites was 2.99 (SD±1.05). This suggests that information on ARBs on most of the evaluated websites was of moderate quality. This analysis is consistent with findings from previous studies which have evaluated the quality of health information on the Internet for a range of different chronic diseases (Table 2) [15, 34-36].

**Table 2:** Comparison of different DISCERN scores for different studies

Topics	Discern score
Bunions [34]	2.9
Hip resurfacing [35]	2.3
General Anesthesia [36]	3.2
Inflammatory bowel disease [15]	3.2

With respect to the quality of health information, most of the websites assessed scored reasonably well in describing the benefits of treatment as well as the risks of using ARBs. Most websites evaluated were also balanced and unbiased, and providing support for shared decision making between patients and physician. This is in agreement with the DISCERN criterion which suggests that a good quality health information resource should include issues for patients to discuss with their healthcare practitioners. However, most of the websites fared poorly in identifying clear sources of information or references, as only five websites met this criterion. This is important because clear sources enable online users examine the credibility and reliability of information on the website or decide to seek further information.

There was consistently a lack of information on the likely consequence of no treatment and how treatment choices affect the overall quality of life. Hence, patients receiving ARB therapy would potentially have poor knowledge on this aspect of their therapy. From the present study, certain websites are Mayo Clinic (Valsartan) 3.84 and Patient Info (Losartan) 3.84, received the highest DISCERN score [31, 32]. These websites were seen to have clearly met a good number of the DISCERN criteria.

The inter-class reliability determined in the study was 0.804 for losartan and 0.695 for valsartan. Ideally a value of 0.7 is considered optimal [37]. Therefore, the values gotten indicated a good level of consistency for quality rating measurements between independent raters. This study highlights that most of the websites had patient information that is potentially difficult to read, as most of the websites were written at readability grade levels higher than grade 8 (Table 3). Of note, the SMOG rating scale was observed to provide a measurement of 2-4 grade levels higher than the Flesch-Kincaid grade (Table 1). This is considered the result of variation between different measurement scales and is consistent with studies conducted by Wilson, 2009 [38]. However, for both scales, most websites consistently scored above “9" indicating the universality of the readability problem.

**Table 3:** Category breakdown of readability scores of ARB websites (n=24)

Readability tools	Category	Number of websites
**FRES**	Easy (80-100)	1
	Average (60-79)	3
	Difficult (0-59)	20
**FKRGL**	Up to grade 6	1
	Grade 6-10	17
	Beyond grade 10	6
**SMOG**	Up to grade 6	-
	Grade 6-10	3
	Beyond grade 10	21

The suboptimal readability observed in the present study is consistent with those of the broader literature. Estrada *et al.*, reported that patient education materials related to the use of anticoagulants were written at grade levels beyond the comprehension of most patients [39]. Hutchinson *et al.*, have also evaluated websites containing medical information pertaining to nine common general medicine diagnoses, and have reported similar readability levels beyond grade 8 for many of the websites evaluated [40]. Our finding is particularly relevant because hypertension is common in the elderly, and its prevalence increases with aging which has been associated with decline in cognitive function [41]. As such, patients on ARBs might find it difficult to read and understand ambiguous online materials. Hence, patient information on these sites should be written at approximately school grade 8 or less to enhance easy understanding. The Institute of Healthcare Improvement recommends using simpler words, shorter sentences and avoiding medical jargon, all of which are important considerations in the provision of online health information for public consumption [42].

### Limitations

The present study is not without a number of limitations. Only the first 50 search results per search engine were reviewed, and only the two mostly prescribed ARBs were assessed. Further, only websites written in English were evaluated. Hence, findings may not be applicable to other languages. The Flesch-Kincaid readability tool used may have overestimated the required readability scores as a list of polysyllabic clinical and medical terms such as ‘angiotensin' and ‘aldosterone' may be regarded as one sentence. Finally, readability formulae used was limited by the lay out of the website page. Websites vary dramatically in how they present information separately from the text that is written. Despite these limitations, the present study provides evidence on suboptimal readability and the need for quality improvement for online ARB patient education material.

## Conclusion

While the overall quality of health information on the selected ARBs is moderate, the websites were found to possess a suboptimal readability score. Future content developers of ARB patient education material should ensure that health information is of favorable quality and easy to read by patients with varying degree of health literacy.

### What is known about this topic

- The internet is an important source of health information;
- Health information on the internet varies widely in quality and a significant number of online health information are written at inappropriate reading levels, which could limit their usefulness.

### What this study adds

- Health information on the reviewed ARBs websites was found to be of moderate quality with written language above recommended reading level;
- Improving the quality of health information available to patients will potentially lead to better treatment outcome.

## Competing interests

The authors declare no competing interests.
